# Supplementary material for: The genome sequence of the Heart and Dart moth, Agrotis exclamationis (Linnaeus, 1758)
Source: Wellcome Open Res. 2024 Oct 2;9:563. [Version 1] doi: 10.12688/wellcomeopenres.23042.1 (PMC11496940; doi:10.12688/wellcomeopenres.23042.1)
Supplement: Peer review history

# Reviewer report: Reviewer response for version 1

**DOI:** 10.21956/wellcomeopenres.25375.r102775

**Published:** 2024-10-25

**Authors:** Savarimuthu Ignacimuthu, Sivasankaran Kuppusamy

**Affiliations:** 1 Manonmaniam Sundaranar University, Tirunelveli, Tamil Nadu, India; 2 Entomology Research Institute, Loyola College, Chennai, Tamil Nadu, India

Authors have done chromosome level genome assembly of *Agrotis exclamationis*. Through the comprehensive genome analysis authors observed 32 chromosomes and identified 20,008 protein-coding genes, and 20,210 gene transcripts. This novel and robust data will be useful for the phylogenomic study of moths.

**Comments on the manuscripts:**

- The taxonomic details “(Arthropoda; Insecta; Lepidoptera;Noctuidae)” of the species need not be mentioned in the abstract
- In the background first paragraph first sentence can be started as “ *Agrotis exclamationis* (Linnaeus, 1758)
- Authors have given the genus name *Agrotis exclamationis *in full form throughout the article. For the first time the genus name can be given in full form, then subsequently it can be given in short form like *A. exclamationis*.
- The last paragraph of the background can be written as “Here we present a chromosomal level genome assembly of *A.exclamationis*, based on a female specimen from the Isle of Bute, Scotland, UK”.
- Mitochondrial genome sequence length 15,392 bp can be included in the text.  
- Above all, I confirm that the manuscript meets the necessary scientific standard and is suitable for indexing"

Are sufficient details of methods and materials provided to allow replication by others?

Yes

Is the rationale for creating the dataset(s) clearly described?

Yes

Are the datasets clearly presented in a useable and accessible format?

Yes

Are the protocols appropriate and is the work technically sound?

Yes

Reviewer Expertise:

Molecular biology and biotechnology

We confirm that we have read this submission and believe that we have an appropriate level of expertise to confirm that it is of an acceptable scientific standard.

# Reviewer report: Reviewer response for version 1

**DOI:** 10.21956/wellcomeopenres.25375.r102783

**Published:** 2024-10-22

**Authors:** Oliver Hawlitschek

**Affiliations:** 1 Department of Evolutionary Biology and Environmental Sciences, University of Zurich, Zurich, Swaziland

The report sounds mostly complete, clear, and straightforward to me. The figures look good to me. I am not an expert on genome sequencing and assembly, so I focused on the organismic part.

 I just have two minor comments:

1. Methods: To match the "Legal and Governance" part, I wonder if it makes sense to describe the legal protection status of the target species in the UK and describe if any permits were necessary.
2. Is there any reason why the two individuals were from two different sites? Could this have any effect on the genome assembly? What happened to ilAgrExcl2?

Kind regards,

 Oliver Hawlitschek

Are sufficient details of methods and materials provided to allow replication by others?

Yes

Is the rationale for creating the dataset(s) clearly described?

Yes

Are the datasets clearly presented in a useable and accessible format?

Yes

Are the protocols appropriate and is the work technically sound?

Yes

Reviewer Expertise:

Phylogenomics and systematics of insects

I confirm that I have read this submission and believe that I have an appropriate level of expertise to confirm that it is of an acceptable scientific standard.

# Reviewer report: Reviewer response for version 1

**DOI:** 10.21956/wellcomeopenres.25375.r103983

**Published:** 2024-10-14

**Authors:** Arun Arumugaperumal

**Affiliations:** 1 Department of Biotechnology, Rajalakshmi Engineering College, Chennai, Tamil Nadu, 602105, India

This is the first report of the whole genome of *Agrotis exclamationis* moth. The first author needs to have an ORCID account. It is very difficult to know additional information about the first author with the address provided. Inside the manuscript text it was mentioned that the first author is associated with Bute Natural History Society. 

 In 'Genome annoatation report' paragraph it was mentioned that there were 1.01 'coding transcripts' per gene. Could the authors elaborate on the reason for this small decimal value observed?

 In Figure 3 lane 4, the x-axis labels are hard to read because of space congestion. The text font size of x-axis label can be reduced to make it more readable.

 A scale could have been included in the photograph of the specimen. The genome was sequenced in a high end PacBio sequencer using the latest technology. The assembly size was 725.10 Mb. The assembly was scaffolded into 32 chromosomes. BUSCO analysis shows a sequence completeness of 98.8% with the database sequence for lepidoptera.

Are sufficient details of methods and materials provided to allow replication by others?

Yes

Is the rationale for creating the dataset(s) clearly described?

Yes

Are the datasets clearly presented in a useable and accessible format?

Yes

Are the protocols appropriate and is the work technically sound?

Yes

Reviewer Expertise:

Bioinformatics

I confirm that I have read this submission and believe that I have an appropriate level of expertise to confirm that it is of an acceptable scientific standard.
